# Auricular Neuromodulation for Mass Vagus Nerve Stimulation: Insights From SOS COVID-19 a Multicentric, Randomized, Controlled, Double-Blind French Pilot Study

**DOI:** 10.3389/fphys.2021.704599

**Published:** 2021-08-02

**Authors:** Claire-Marie Rangon, Régine Barruet, Abdelmadjid Mazouni, Chloé Le Cossec, Sophie Thevenin, Jessica Guillaume, Teddy Léguillier, Fabienne Huysman, David Luis

**Affiliations:** ^1^Pain and Neuromodulation Unit, Neurosurgery Department, Hôpital Fondation Adolphe de Rothschild, Paris, France; ^2^Infectious Diseases Department, Centre Hospitalier Simone Veil, Beauvais, France; ^3^Pain Unit, Centre Hospitalier Simone Veil, Beauvais, France; ^4^Clinical Research Department, Hôpital Fondation Adolphe de Rothschild, Paris, France; ^5^Clinical Research Department, Centre Hospitalier Simone Veil, Beauvais, France; ^6^Intensive Care Unit, Centre Hospitalier Simone Veil, Beauvais, France

**Keywords:** auricular neuromodulation, vagus nerve stimulation, COVID-19, pandemics, NF-κB, cholinergic anti-inflammatory pathway, non-invasive neuromodulation

## Abstract

**Importance:** An exacerbated inflammatory response to severe acute respiratory syndrome coronavirus 2 (SARS-CoV-2) infection is believed to be one of the major causes of the morbidity and mortality of the coronavirus disease 2019 (COVID-19). Neuromodulation therapy, based on vagus nerve stimulation, was recently hypothesized to control both the SARS-CoV-2 replication and the ensuing inflammation likely through the inhibition of the nuclear factor kappa-light-chain-enhancer of activated B cells pathway and could improve the clinical outcomes as an adjunct treatment. We proposed to test it by the stimulation of the auricular branch of the vagus nerve, i.e., auricular neuromodulation (AN), a non-invasive procedure through the insertion of semipermanent needles on the ears.

**Objective:** The aim of this study was to assess the effect of AN on the clinical outcomes in patients affected by COVID-19.

**Design, Setting, and Participants:** A multicenter, randomized, placebo-controlled, double-blind clinical trial included 31 patients with respiratory failure due to COVID-19 requiring hospitalization. Within 72 h after admission, patients received either AN (*n* = 14) or sham neuromodulation (SN, *n* = 15) in addition to the conventional treatments.

**Main Outcome and Measures:** The primary endpoint of the study was the rate of a clinical benefit conferred by AN at Day 14 (D14) as assessed by a 7-point Clinical Progression Scale. The secondary endpoint of the study was the impact of AN on the rate of transfer to the intensive care unit (ICU) and on the survival rate at D14.

**Results:** The AN procedure was well-tolerated without any reported side effects but with no significant improvement for the measures of both primary (*p* > 0.3) and secondary (*p* > 0.05) endpoints at the interim analysis. None of the AN-treated patients died but one in the SN group did (81 years). Two AN-treated patients (73 and 79 years, respectively) and one SN-treated patient (59 years) were transferred to ICU. Remarkably, AN-treated patients were older with more representation by males than in the SN arm (i.e., the median age of 75 vs. 65 years, 79% male vs. 47%).

**Conclusion:** The AN procedure, which was used within 72 h after the admission of patients with COVID-19, was safe and could be successfully implemented during the first two waves of COVID-19 in France. Nevertheless, AN did not significantly improve the outcome of the patients in our small preliminary study. It is pertinent to explore further to validate AN as the non-invasive mass vagal stimulation solution for the forthcoming pandemics.

**Clinical Trial Registration:** [https://clinicaltrials.gov/], identifier [NCT04341415].

## Introduction

The coronavirus disease 2019 (COVID-19) pandemic has overwhelmed the sanitary capacity. Additional therapeutic arsenals that could reduce the morbidity rate, although untested in the given context but previously proven to be efficacious in a related clinical context, are urgently needed. The role of the nervous system in respiratory failure in patients with COVID-19 has been recently emphasized (Li et al., 2020; Manganelli et al., 2020; Tassorelli et al., 2020). The heavy viral infection within the brain stem of deceased patients suggests that the neuroinvasive potential of SARS-CoV2 is likely to be partially responsible for COVID-19 acute respiratory failure. This finding favors treatment modalities involving the vagus nerve and the cholinergic anti-inflammatory pathway (CAP), which was supported by several research teams (Bara et al., 2020; Bonaz et al., 2020; De Virgiliis and Di Giovanni, 2020; Leitzke et al., 2020; Mazloom, 2020; Pomara and Imbimbo, 2020; Rangon et al., 2020; Staats et al., 2020; Tornero et al., 2020; Azabou et al., 2021; Mastitskaya et al., 2021).

In fact, the key role of the vagus nerve in controlling inflammation through the so-called “inflammatory reflex” was highlighted almost 20 years ago by Tracey (Tracey, 2002), with the concept constantly being refined, particularly with the description of the CAP (Czura et al., 2003; Pavlov and Tracey, 2005; Oke and Tracey, 2008; Andersson and Tracey, 2012; Olofsson et al., 2012; Pereira and Leite, 2016; Chavan and Tracey, 2017; Serhan et al., 2018, 2019; Bonaz, 2020a). It is now well-acknowledged that the immune-inflammatory processes are modulated by the vagus nerve in a significant manner. Therefore, the vagus nerve modulation appears to be a good candidate to tackle the COVID-19-associated cytokine storm.

In fact, in animal models, it was demonstrated that the stimulation of the vagus nerve modulates immune response through the nuclear factor kappa-light-chain-enhancer of activated B cells (NF-κB) pathway (O'Mahony et al., 2009; Sun et al., 2013; Leitzke et al., 2020). NF-κB, a family of evolutionarily conserved transcription factors, is a double-edged sword capable of inducing the expression of both antiviral host factors and viral genes in a context-dependent manner (Deng et al., 2018). It has previously been shown that the respiratory syncytial virus (RSV) (Masaki et al., 2011), the porcine reproductive and respiratory syndrome virus (PRSSV) (Wang et al., 2013), and a variety of coronaviruses were shown to divert the NF-κB to the benefit of their own replication (Poppe et al., 2017). Recently, the hyperactivation of the NF-κB pathway has been implicated in the pathogenesis of severe/critical COVID-19 phenotype (Hariharan et al., 2020; Hirano and Murakami, 2020). Thus, the modulation of the NF-κB pathway in favor of host defense, through vagus nerve stimulation (VNS), is particularly attractive against viral infections.

Interestingly, the vagal tone, which modulates the activity of the inflammatory reflex in humans, can be monitored through the measurement of the heart rate variability (HRV) (Thayer, 2009; Williams et al., 2019). The HRV constitutes a physiological marker of the vagal tone, quick to measure and non-invasive, due to the continuously monitored ECG or even an assessment by a handy smartphone (Chen et al., 2020; Shaffer et al., 2020).

Several epidemiological studies have shown that reduced HRV is a risk factor for all-cause mortality and morbidity (Liao et al., 2002), not only in cardiovascular diseases (Fang et al., 2020), metabolic diseases (Pavlov, 2021), and neurodegenerative diseases (Rangon et al., 2020), but also in acute respiratory distress syndrome (ARDS) (Chen et al., 2018), sepsis (De Castilho et al., 2018; Barnaby et al., 2019), and the severe infection of severe acute respiratory syndrome coronavirus 2 (SARS-CoV-2) (Hasty et al., 2020; Leitzke et al., 2020; Aragon-Benedi et al., 2021; Pan et al., 2021).

Given this, an ideal therapeutic “all-in-one” approach for the COVID-19 pandemic should be able to (1) increase the HRV significantly, (2) be readily operational, (3) target not only the “regular” SARS-CoV-2 but also the current and emerging virulent variants, (4) provide minimal adverse events given the frailty of the target population, and (5) be less time-consuming and cost-effective.

Vagus nerve stimulation is presently achieved either by pharmacological or by neuromodulatory approaches. Regrettably, the non-pharmacological therapeutic strategies that target the immune-inflammatory processes and thereby could potentially improve the outcome of the patients with COVID-19 have not so far been sufficiently highlighted (Azabou et al., 2021). In fact, the vagus nerve holds a specific and important area in bioelectronic medicine, an evolving field, in helping diagnosis and treatment of the disease (Pavlov et al., 2020).

Invasive VNS (iVNS), using a specifically designed surgically implantable electrode cuff (for selective activation of the CAP) wrapped around the cervical vagus nerve, has recently been suggested for neuroimmunomodulation in COVID-19 (Mastitskaya et al., 2021). Nevertheless, implanting a VNS device in patients who are critically ill can be challenging, and hence, non-invasive transcutaneous VNS received particular attention as evidenced by the launch of several clinical trials (i.e., NCT04368156, NCT04379037, NCT04382391, NCT04638673, and NCT04514627). Among the two options, one is transcutaneous cervical VNS (tcVNS), where the stimulating electrodes are applied to the skin surface over the sternocleidomastoid muscle in the neck, and the other, the auricular VNS (taVNS or aVNS), which targets the auricular branch of the vagus nerve (ABVN) that innervates part of the skin of the outer ear, mainly the auricular concha and most of the area around the auditory meatus (Peuker and Filler, 2002; Butt et al., 2020). Both tcVNS and taVNS have been shown to elicit comparable therapeutic effects as VNS (for a review, Yap et al., 2020).

Remarkably, taVNS, the bioelectronic medicine approach targeting only the afferent arm of the vagus nerve, makes it easier to read into HRV, a marker for efferent vagal activation (Burger et al., 2020). Besides, the taVNS was proven not only to modulate the activity of NF-κB in animal models (Zhao et al., 2012) but also to increase the HRV values in healthy humans, to reduce sepsis, and to increase the survival rate, both significantly, in experimental models (for a review, Rangon et al., 2020). Hence, the taVNS is opted in four out of the five ongoing clinical trials assessing the impact of bioelectronic non-invasive VNS in COVID-19 (i.e., NCT04379037, NCT04382391, NCT04638673, and NCT04514627). Nevertheless, the bioelectronic approach, which is still in the development phase, requires additional investigations to establish the parameters for optimum stimulation, especially in the crucial pandemic situation (Bonaz, 2020b; De Virgiliis and Di Giovanni, 2020).

As we are overwhelmed (more than 3 million deaths worldwide) by the present context, also potentially in the near future by more and more emerging virulent variants, it is time to take advantage of scientifically assessed complementary medicine that has the benefit of having some hindsight. From 2009, Tracey highlighted the need to learn from the acquired knowledge in acupuncture technics (Oke and Tracey, 2009). In fact, in 2021, VNS through acupuncture was suggested as a feasible approach to activate the CAP to control the COVID-19-associated inflammatory burst (Qin et al., 2021).

Interestingly, the non-electrical stimulation of the ABVN through auricular acupuncture or acupressure, using either needles, seeds, or beans, is also able to increase the HRV, both in rats (Gao et al., 2012a) and in humans (Hsu et al., 2007; Gao et al., 2012b; Arai et al., 2013). It is quite understandable that the physical stimulation of the external acoustic meatus (innervated by the ABVN) has been known since the nineteenth century to elicit a cough reflex induced by vagal regulation, the so-called “Arnold's reflex” (He et al., 2012).

**Figure 1:**
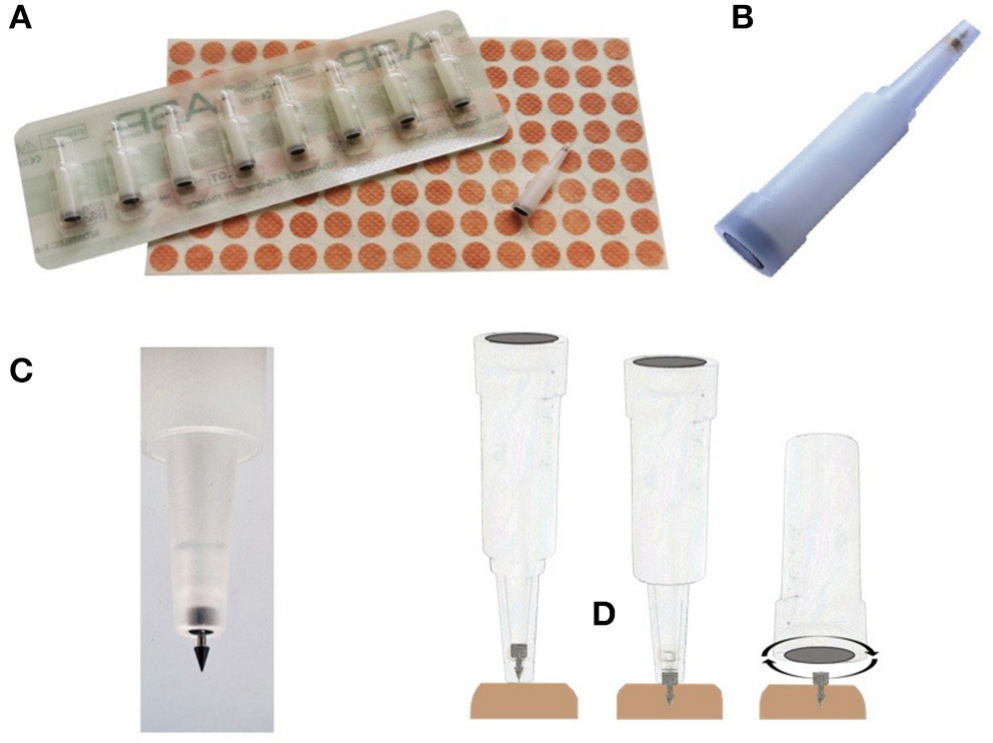
The semipermanent needles (SPNs). **(A)** SPN packaging. **(B)** Close-up of a SPN inside its sterile blister. **(C)** Close-up of a SPN ready to be inserted in the skin of the outer ear. **(D)** The three-step procedure for SPN insertion: (1) the blister is placed on the disinfected skin point, (2) a pressure exerted on the blister delivers the SPN inside the skin, and (3) the SPN is once again sinked using the bottom end of the blister (images from Sedatelec^R^, Irigny, France).

Given this, auricular acupuncture using semipermanent needles (SPNs, Figure 1), i.e., auricular neuromodulation (AN), can be conceived to provide sustained and personalized vagus stimulation. The rationale resides in the histology of acupoints that of course exhibits intersubject variability (i.e., age, gender, diseases, etc.). These acupoints have relatively lower electrical impedance than the non-acupoints, the former further depending on the architecture of the so-called “neurovascular complex,” which is formed by a combination of myelinated and unmyelinated nerve fibers, small arterial and venous capillaries, and a small lymphatic vessel (Rabischong and Terral, 2014). Thus, the SPN remains on the acupoints of the ears (from a few days to several weeks) and falls spontaneously, depending on each individual.

Therefore, the protocol of taVNS with SPN is a “ready-to-go” procedure with no need of presetting the parameters of stimulation (Bonaz, 2020b), and it is a rapid one (i.e., it takes <10 s to put one SPN on the ear, Figure 1D). The only parameters to determine on the spot are the number and the localization of the SPNs on the ear. Such user-friendliness may explain the enthusiasm of the American soldiers on the battlefield to use five SPNs per ear directly to alleviate severe acute pain in the past 20 years (Niemtzow, 2018). Studies on the mechanism of action suggested that afferent projections from the ABVN to the nucleus of the solitary tract constitute the anatomical basis for the vagal regulation and the analgesic effects in the battlefield (i.e., auricular) of acupuncture (He et al., 2012; Usichenko et al., 2017). Since the COVID-19 pandemic has often been metaphorically represented as a war situation, auricular vagus nerve modulation with SPN is very likely to be successfully implemented in the emergency department or at the clinic (Niemtzow, 2020).

Due to its role in modulating NF-κB pathway, AN could lead to a decrease in the host inflammatory response and to a decrease in the SARS-COV-2 replication. Thus, modulation of this signalling pathway could result in both a decrease in the number of admissions to intensive care units (ICU) and a decrease in mortality (Li et al., 2020). In this pilot trial, we investigated the impact of AN through SPNs on the short-term outcome (i.e., 14 days) of inpatients with respiratory failure due to COVID-19.

## Materials and Methods

The study followed the CONSORT checklist (http://www.consort-statement.org).

The study was approved by the CPP SUD-Est II, an ethical board affiliated with the French Ministry of Health.

### Patients and Treatments

Adults of both sex (over 18 years old) with confirmed COVID-19 ARDS, based on the positive PCR for SARS-CoV2 and/or a suggestive chest scan and the following clinical criteria (i.e., abnormal lung auscultation OR SpO2 <94% OR oxygen supplementation OR non-invasive ventilation), who were admitted to the Hôpital Fondation Adolphe de Rothschild (Paris) or the Hôpital Simone Veil (Beauvais, France) were proposed, within the first 72 h, to participate in the randomized controlled double-blind trial. In case of cognitive disorders or measures of legal protection, informed consent was obtained by phone from a trustworthy person designed in the medical file. Pregnant or breastfeeding women were excluded. Former critical inpatients could secondarily be included within 72 h after their transfer to a non-ICU.

During the first semester of 2020, there were not enough data available in the literature to calculate the number of patients needed to treat. Thus, 60 patients (30 per arm) were planned for inclusion in our pilot study. If the results proved to be interesting, they would allow to calculate the number to treat secondarily to set a broader trial. A futility intermediate analysis was planned at midcourse, allowing the study to continue, provided that the *P*-value was inferior to 0.3 in favor of the verum group.

**Figure 2:**
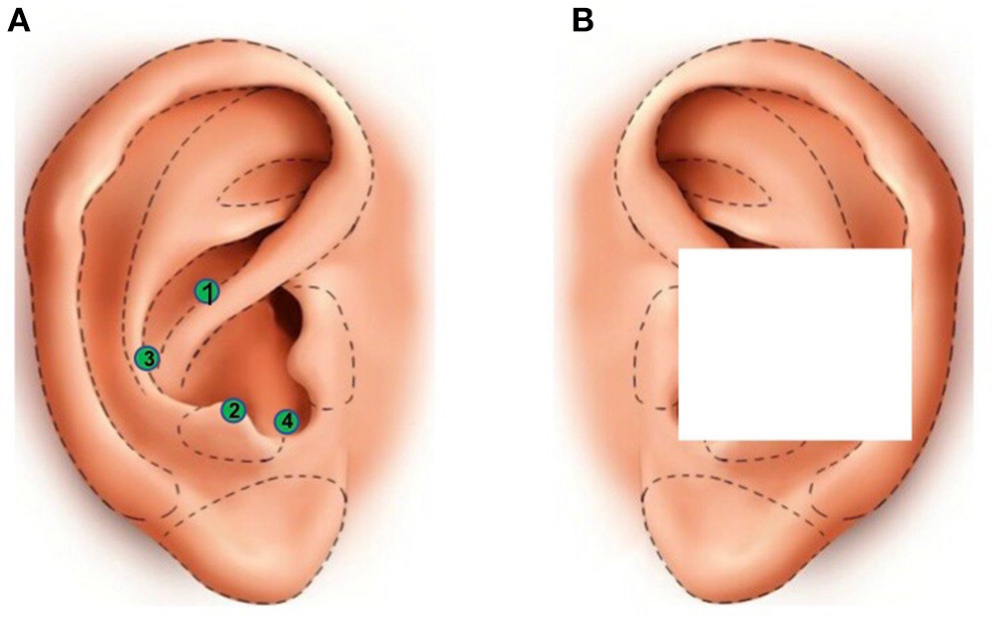
Neuromodulation with SPNs on the concha. **(A)** Localization of the four selected acupoints, represented as green circles, of the concha, following the order indicated by the number in the circle: (1) master point of endoderm, (2) master point of reticular formation, (3) thymic plexus, and (4) ACTH point (i.e., ear maps according to Alimi, 2017). Each acupoint is either implanted with SPNs (verum group) or pressed on with the empty needle applicator (sham group). **(B)** The white square symbolizes the opaque Band-Aid allowing double-blind treatment. Both ears of the patients receive same neuromodulation treatment (i.e., verum or sham group). With the permission of Sedatelec^R^.

Patients were randomized to receive either verum auricular vagus nerve (AN) or sham (SN) neuromodulation. After disinfection of the ears with Chlorhexidine^R^, the trained physician wearing gloves carried out neuromodulation treatment, beginning with the right ear and then the left ear of the patient, without the presence of the nursing staff in the room. In the verum group, four semipermanent sterile needles (Classic ASP, Sedatelec^R^, Irigny, France) were implanted on each auricle, i.e., eight needles per patient, following an order and a precise localization (surrounding the concha): upside, bottom side, external side, and internal side, corresponding to the following acupoints: (1) master point of endoderm, (2) master point of reticular formation, (3) thymic plexus, and (4) adrenocorticotropin hormone (ACTH) point (ear maps according to Alimi, 2017; Figure 2A). Hydrogen peroxide (Herouville Saint Clair, France) was then applied on each implanted SPN to stop potential bleeding. No electrical stimulation was subsequently applied on the needles. The concha was subsequently hidden by an opaque and waterproof Band-Aid (i.e., made with a compress under a Tegaderm^R^, fixed thanks to Steril-Strips^R^, Figure 2B). In the sham group, no needle was implanted. Instead, the trained physician pressed the empty needle applicator against the four acupoints of each concha before placing the opaque Band-Aid. In fact, the pressure applied on each auricle with the needle applicator on the selected acupoints was constantly painful by itself, and then, it could yield the same subjective perception as true needles to the patients with hypoxic-ischemic encephalopathy. Moreover, undertaking the sham stimulation outside the auricular region innervated by the ABVN, such as the ear lobe, called “location sham,” was recently advised against (Rangon, 2018; Borges et al., 2021; Verma et al., 2021). The patients, their physicians, and nursing staff were blind about randomization because the ears were hidden by the opaque Band-Aid throughout the hospitalization. Patients of both groups received the regular drugs used for COVID-19 pneumonia (i.e., corticosteroids, antibiotics, etc.) in accordance with the current practice guidelines at the hospital at that time, in addition to neuromodulation treatment. An industrial partner (Sedatelec^R^) has proposed to provide the SPNs for the procedure to run this clinical trial.

### Clinical Status Assessment

Clinical status was assessed using a 7-category ordinal scale (Cao et al., 2020): (1) not hospitalized with the resumption of normal activities, (2) not hospitalized, but unable to resume normal activities, (3) hospitalized, not requiring supplemental oxygen, (4) hospitalized, requiring supplemental oxygen, (5) hospitalized, requiring nasal high-flow oxygen therapy, non-invasive mechanical ventilation, or both, (6) hospitalized, requiring extracorporeal membrane oxygenation (ECMO), invasive mechanical ventilation, or both, and (7) death.

1. not hospitalized with the resumption of normal activities;
2. not hospitalized, but unable to resume normal activities;
3. hospitalized, not requiring supplemental oxygen;
4. hospitalized, requiring supplemental oxygen;
5. hospitalized, requiring nasal high-flow oxygen therapy, non-invasive mechanical ventilation, or both;
6. hospitalized, requiring extracorporeal membrane oxygenation (ECMO), invasive mechanical ventilation, or both; and
7. death.

### Laboratory Tests

The collection of biological assessments [in particular, C-reactive protein (CRP) blood tests] and imaging were decided by the physicians of the COVID unit (this protocol does not). Ethics approval for analysis of all the data collected was waived by the hospital Institutional Review Board since all the data of the patients collected conformed to the policies.

### Statistical Analysis

Statistical analyses were performed using the R software (version 4.0.3). The descriptive analyses of the qualitative variables were presented as number and percentage and that of the quantitative variables were presented as mean and SD. Two means of quantitative variables were compared using the Student's *t*-test if the assumptions were verified, if not the Wilcoxon–Mann–Whitney non-parametric test was used. The time until clinical improvement and the CRP at recruitment was analyzed using the Wilcoxon–Mann–Whitney test, and the age at recruitment was analyzed using the Student's *t*-test. For all the comparisons of categorical variables, the Fisher's exact test was used since the assumption for the Pearson's chi-square test was not valid for any of them. All statistical tests were two-sided, and the significance level fixed for interim analyses was 30%.

## Results

### Patient Characteristics

**Figure 3:**
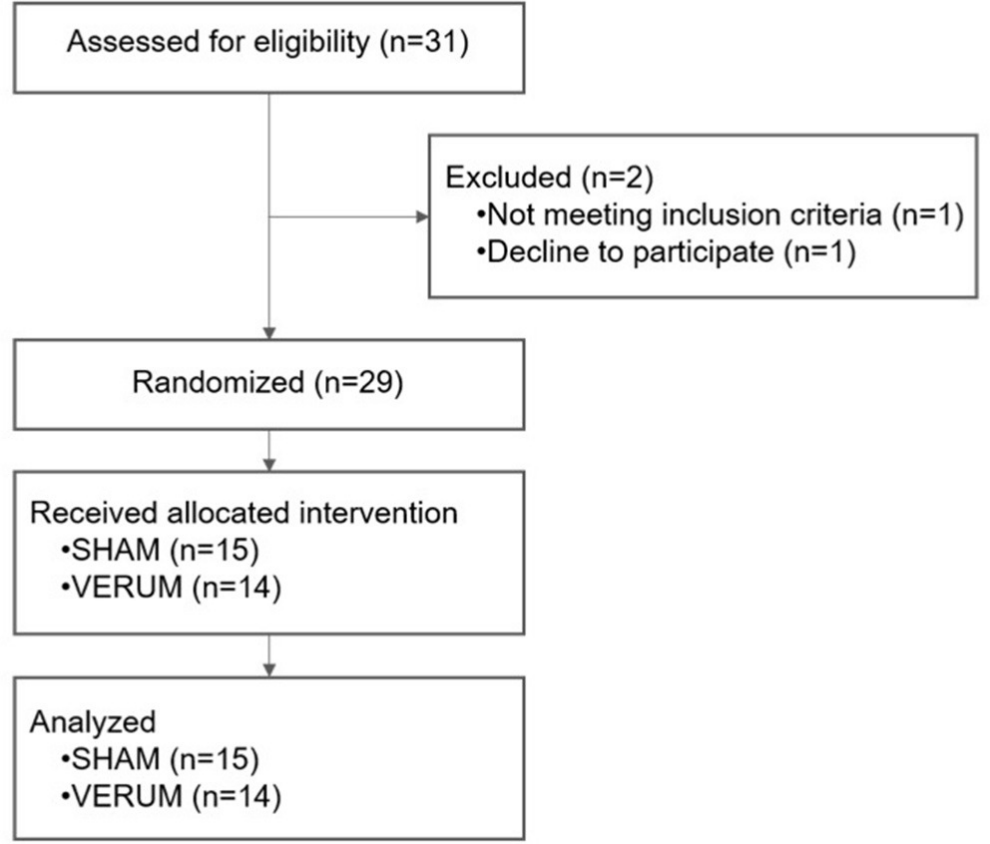
Study flowchart.

Thirty-one patients with respiratory failure due to COVID-19 requiring hospitalization for non-invasive oxygen supplementation were included in this study. Among them, 29 have been analyzed (i.e., study flowchart, Figure 3). Within 72 h after admission, patients received either auricular (verum group, *n* = 14) or placebo neuromodulation therapy (sham group, *n* = 15) in addition to their usual treatments. The sham group was composed of 7 males and 8 females (mean age = 68.5 ± 15.6 years), and the verum group was composed of 11 males and 3 females (mean age = 73.4 ± 11.6 years). Whereas, there was no statistically significant difference between sham and verum groups regarding mean age (Table 1), the median age of the AN arm was roughly 10 years old (75.5 vs. 65 years). Moreover, gender was not distributed in a balanced way between both groups, with 47 and 79% of males in the sham and the verum group, respectively. Regardless of the group considered, all patients required oxygen therapy with one patient requiring mechanical ventilation in the Sham group. Regarding CRP levels, all patients presented a proinflammatory state, and no statistical difference between groups was observed for this parameter.

**Table 1:** Characteristics of study patients at recruitment.

**Variables**	**SHAM (*n* = 15)**	**VERUM (*n* = 14)**	***P***
Gender, male, *n* (%)	7 (47)	11 (79)	0.17^a^
Age (mean ± SD, years)	68.5 ± 15.6	73.4 ± 11.6	0.34^b^
Age (median, years)	65.0	75.5	
Initial hospitalization in a recruiting center, *n* (%)	9 (60)	7 (50)	0.87^a^
Oxygen therapy required, *n* (%)	14 (93)	14 (100)	1^a^
High flow nasal oxygen or MV required, *n* (%)	1 (6.7)	0 (0)	
CRP (mean ± SD, mg/L)	71.0 ± 43.1	89.96 ± 77.5	0.77^c^
**Comorbidities**			
Hypertension (%)	8 (47)	8 (57)	0.40^c^
Diabetes (%)	8 (47)	5 (36)	0.78^c^
Asthma (%)	0 (0)	2 (14)	
Dyslipidemia (%)	4 (24)	2 (14)	0.54^c^
Renal disease (%)	2 (12)	0 (0)	
Cardiovascular disease (%)	2 (12)	1 (7)	0.93^c^
Vascular disease (%)	2 (12)	3 (21)	0.49^c^
Obesity (%)	4 (24)	3 (21)	0.91^c^

*CRP, C-reactive protein; MV, mechanical ventilation.*

a *Fisher's exact test*.

b *Student's t-test*.

c *Wilcoxon–Mann–Whitney test*.

### Patient Clinical Evolution

We first evaluated the effects of AN inpatient outcomes regarding the 7-category ordinal scale evolution or hospital discharge. Concerning these parameters, we did not observe any clinical improvement in the verum group compared to the sham group (Table 2). Even if we adjusted these parameters to gender, age, or CRP levels, no statistical difference was found between study groups (i.e., data not shown). We then evaluated the time until clinical improvement and the transfers to the ICU for the study patients. Again, there was no statistically significant difference between groups. Finally, for the 29 patients in both groups, one case in the sham group died.

**Table 2:** Clinical evolution of patients during the medical follow-up.

**Outcome measures**	**SHAM (*n* = 15)**	**VERUM (*n* = 14)**	***P***
7-category ordinal Clinical Progression Scale score improvement or Hospital discharge, *n* (%)	12 (80)	9 (64)	0.43^a^
Time until clinical improvement (mean ± SD, days)	8.8 + 3.5	8.9 + 3.3	0.86^c^
Transfers to ICU, *n* (%)	1 (6.7)	2 (14.3)	0.60^a^
14-day all-cause mortality, *n* (%)	1 (7)	0 (0)	1^a^

a *Fisher's exact test.*

b *Student's t-test*.

c *Wilcoxon–Mann–Whitney test*.

## Discussion

The COVID-19 infection has rapidly spread throughout the world causing a major healthcare crisis. About 20% of patients with COVID-19 develop severe disease requiring hospitalization. Among them, a high mortality rate of up to 97% is observed with respiratory failure as the leading cause of death. Despite many therapeutic strategies under investigation, there is still no curative treatment available. With the increasing rates of infection, there is an urgent need for new therapeutic approaches to counteract the infection. The excessive inflammatory response to SARS-CoV-2 is thought to be a major cause of acute respiratory failure in those patients. As the nervous system has shown to be a strong modulator of respiratory function and the immune response, we suggested as others that neuromodulation could be used to improve patient outcomes. As a result, we raised the hypothesis that AN could be used as a potential adjunct treatment to modulate inflammatory response in patients with COVID-19 and improve their clinical outcomes. Thus, we explored the clinical effects of AN in patients with COVID-19 using a 7-category ordinal Clinical Progression Scale (Cao et al., 2020), or hospital discharge as primary study outcome and the transfers to the ICU and survival rate as secondary study outcomes.

In our pilot study, patients of both arms are likely to be as severely infected by SARS-CoV-2, as shown by the identical median of the CRP (Deng et al., 2020; Terpos et al., 2020).

Regardless of the endpoint considered, AN does not appear to improve inpatients with COVID-19 outcomes.

This result could be explained by three major limitations. First, we hypothesized that taVNS was able to improve the patient outcome by modulating the excessive inflammatory response due to SARS-CoV-2 infection. However, we failed to show any anti-inflammatory effect of AN using CRP as a biomarker. Other more specific markers such as tumor necrosis factor-alpha (TNF-α) or interleukin-1 beta (IL-1β) should be used in further studies to highlight the potent anti-inflammatory effects of AN in those patients but would imply additional invasive assessment for the patient.

Second, age and gender differences in immune responses have been reported in infectious diseases such as COVID-19 with more elderly men than young women dying from the disease. By pure coincidence (due to non-paired randomization), AN and PN small population are not set on equal footing regarding COVID-19 prognosis factors. Therefore, our two groups are evidently not comparable regarding age and gender, while those remain comparable regarding comorbidities (Table 1), CRP (Table 1), and smoking status (i.e., all the patients included were non-smokers). The age issue is considered as the strongest prognosis factor for COVID-19 (Izcovich et al., 2020; O'Driscoll et al., 2021). Our study is in line with this result, as the improvement in the respiratory status of the patients of both arms is significantly correlated to their age (*p* < 0.03). Considering that the case fatality rate of the 70- to 79-year-old patients was shown to be roughly two times higher than the case fatality rate of the 60- to 69-year-old patients (Signorelli and Odone, 2020), whatever the country considered (Chen et al., 2021), the AN group, showing a median age 10 years older than the PN group (75.5 vs. 65 years old), was expected to count more deaths than the placebo arm. Moreover, in our study, 80% of the verum population consisted of men, contrary to the sham group (i.e., <50%). Considering that the male gender is correlated to a bad outcome in the COVID-19 pandemic, it is noteworthy that there are fewer deaths in the AN arm than in the placebo arm. Such age and gender differences between groups may represent the major source of bias in our study, and in particular given the small size.

Third, we acknowledged that our cohort is too small to draw any firm conclusion, but this preliminary study provides some leads and merits further exploration with a much larger study population to assess if AN-reduced inflammation could confer potential health benefits to the patients with COVID-19. In fact, the results of our pilot study provide us clues to optimize the design of the relatively larger upcoming clinical trials.

In fact, first, the choice of primary and secondary endpoints can be better defined by this experience. In the chaotic situation of the first COVID-19 wave, we wanted to select the most convenient primary endpoint, which is easy to acquire from medical files, i.e., the clinical improvement on a pragmatic validated scale (Cao et al., 2020). Nevertheless, contrary to the known effect of fast pain relief, taVNS through SPN might require a relatively longer period (i.e., more than 14 days) to elicit a significant clinical improvement in severe SARS-CoV-2 infection (as suggested by Pan et al., 2021). In fact, iVNS is known to be a slow-acting therapy as reported in epilepsy (Panebianco et al., 2015) and inflammatory bowel diseases (Sinniger et al., 2020). It might have been more relevant to assess the mortality rate at Day 28 (D28) instead of Day 14 (D14) (Cao et al., 2020; Hermine et al., 2021)), as well.

In contrast, choosing the rate of transfer to the ICU as a primary endpoint might have been more appropriate, as it generally happens within 10 days after hospital admission (Cheng et al., 2020). Nevertheless, contrary to the Beauvais General hospital, the Rothschild Foundation Hospital is not a frontline hospital for patients with COVID-19, only admitting transfers from other centers. Therefore, a significant percentage (i.e., half) of our small population might not be suitable for assessing AN efficiency in COVID-19.

Besides fatality and the rates of transfer to the ICU, HRV increase should be selected as a primary endpoint, allowing a more discerning assessment of AN efficiency with small patient samples. In fact, HRV eases bias induced by comorbidities, age, gender, drugs, and nowadays vaccines since the latter factors influence the basal HRV values (Hasty et al., 2020; Wee et al., 2020). Regrettably, in this pilot study, we did not monitor vagal tone through HRV. Such monitoring is not at all cumbersome as the patients are continuously monitored. In fact, in a recent study, a Holter monitor was used to record, collect, and analyze the dynamic ECG data over 24 consecutive hours for all 34 patients (Pan et al., 2021). Nevertheless, most non-ICU patients were regularly, but only occasionally, monitored, and the medical-grade accuracy of HRV by the smartphone apps was not available at that time in our hospitals. However, we believed that the HRV parameter constitutes an optimal primary endpoint for the upcoming trials (Pan et al., 2021).

Second, AN should be dispensed earlier. As AN does not elicit noteworthy side effects, patients should receive AN as soon as they are presumed to be COVID-19 positive, ideally at the clinic, for instance, long before receiving the PCR results of SARS-CoV-2, or at least upon arrival at the emergency ward. This time-saving process would help decrease the replication of the virus and the inflammatory response.

Finally, AN efficiency might be optimized regarding the choice of the localization of the SPNs on the ear. In particular, one localization has drawn attention, i.e., outside of the ear concha (Volf et al., 2020). The main barrier lies in the fact that double-blinding gets more difficult, requiring a larger Band-Aid, less easy and comfortable to wear for patients during several days.

## Conclusion

Auricular neuromodulation with Semi Permanent Needles was successfully implemented in two hospitals during the COVID-19 pandemic and was well-tolerated by oxygen-requiring patients with COVID-19. However, in our preliminary study, this non-invasive vagus nerve neuromodulation technique did not significantly improve the outcome of the patients with COVID-19 when applied within the first 72 h of hospitalization. Other studies are necessary to clarify these results and to assess if reduced inflammation induced by AN is sufficient to induce potential health benefits in those patients.

Contrary to conventional approaches, autonomic neuroimmunology, whereby immune functions can be modulated by the vagus nerve, targets a common hallmark of immune dysregulation across infectious diseases and improves the homeostasis potential of the host. As the vagally driven CAP can stop the action of NF-κB, adequate vagal signaling might modulate the severity of several viral infections, thus supporting complementary non-invasive vagal neuromodulation use in one-size-fits-all antiviral strategy, now in case of vaccine shortage or poor efficiency or after, for the upcoming pandemics.

## Data Availability Statement

The raw data supporting the conclusions of this article will be made available by the authors, without undue reservation.

## Ethics Statement

The studies involving human participants were reviewed and approved by CPP SUD-Est II affiliated to the French Ministry of Health. The patients/participants provided their written informed consent to participate in this study.

## Author Contributions

C-MR, the principal investigator, did all the neuromodulation treatments (auricular and sham treatments) in Hôpital Fondation Rothschild and part of them in Centre Hospitalier de Beauvais and wrote the manuscript. RB and DL selected the patients of Beauvais Hospital for inclusion. AM did some part of the neuromodulation treatments in Beauvais Hospital. ST coordinated the study between the two hospitals (Head project). CL, TL, and FH collected and analyzed the data from medical records. JG and CL did the statistical analysis. DL made it possible to have a multi centric center (Director of Clinical Research Department in Beauvais Hospital). All authors contributed to the article and approved the submitted version.

## Conflict of Interest

SEDATELEC gave financial support. The authors declare that the research was conducted with conflict of interest.

## Publisher's Note

All claims expressed in this article are solely those of the authors and do not necessarily represent those of their affiliated organizations, or those of the publisher, the editors and the reviewers. Any product that may be evaluated in this article, or claim that may be made by its manufacturer, is not guaranteed or endorsed by the publisher.

## Abbreviations

ABVN: Auricular Branch of the Vagus Nerve
ACTH: Adrenocorticotropin Hormone
ANS: Autonomic Nervous System
AN: Auricular Neuromodulation
ARDS: Acute Respiratory Distress Syndrome
CAP: Cholinergic Anti-inflammatory Pathway
COVID-19: Coronavirus Disease 2019
CRP: C-Reactive Protein
HRV: Heart Rate Variability
ICU: Intensive Care Unit
NF-κB: Nuclear Factor Kappa-light-chain-enhancer of activated B cells
SARS-CoV-2: Severe Acute Respiratory Syndrome Coronavirus 2
SN: Sham Neuromodulation
SPN: Semipermanent Needle
VNS: Vagus Nerve Stimulation
tVNS: transcutaneous VNS
taVNS: transcutaneous auricular VNS
tcVNS: transcutaneous cervical VNS.
